# Mesenchymal stem cells alleviate experimental autoimmune cholangitis through immunosuppression and cytoprotective function mediated by galectin-9

**DOI:** 10.1186/s13287-018-0979-x

**Published:** 2018-09-17

**Authors:** Junyu Fan, Xiaojun Tang, Qian Wang, Zhuoya Zhang, Shufang Wu, Wenchao Li, Shanshan Liu, Genhong Yao, Hongwei Chen, Lingyun Sun

**Affiliations:** 1 Nanjing Drum Tower Hospital, Clinical College of Traditional Chinese and Western Medicine, Nanjing University of Chinese Medicine, Nanjing, 210008 China; 2 Department of Rheumatology and Immunology, The Affiliated Drum Tower Hospital of Nanjing University Medical School, 321 Zhongshan Road, Nanjing, 210008 China

**Keywords:** Umbilical cord–derived mesenchymal stem cells, Primary biliary cholangitis, Galectin-9, Inflammation

## Abstract

**Background:**

Mesenchymal stem cells (MSCs) play an anti-inflammatory role by secreting certain bioactive molecules to exert their therapeutic effects for disease treatment. However, the underlying mechanism of MSCs in chronic autoimmune liver diseases—primary biliary cholangitis (PBC), for example—remains to be elucidated.

**Methods:**

Human umbilical cord–derived MSCs (UC-MSCs) were injected intravenously into 2-octynoic acid coupled to bovine serum albumin (2OA-BSA)-induced autoimmune cholangitis mice. Serum levels of biomarkers and autoantibodies, histologic changes in the liver, diverse CD4^+^ T-cell subsets in different tissues, and chemokine activities were analyzed. Moreover, we investigated galectin-9 (Gal-9) expression and its function in UC-MSCs.

**Results:**

In this study, UC-MSC transplantation (UC-MSCT) significantly ameliorated liver inflammation, primarily by diminishing T helper 1 (Th1) and Th17 responses as well as modifying liver chemokine activities in experimental autoimmune cholangitis mice. Mechanistically, UC-MSCs significantly repressed the proliferation of CD4^+^ T cells and suppressed the differentiation of Th1 and Th17 cells, which was likely dependent on Gal-9. Furthermore, the signal transducer and activator of transcription (STAT) and c-Jun N-terminal kinase (JNK) signaling pathways were involved in the production of Gal-9 in UC-MSCs.

**Conclusions:**

These results suggest that Gal-9 contributes significantly to UC-MSC–mediated therapeutic effects and improve our understanding of the immunomodulatory mechanisms of MSCs in the treatment of PBC.

**Electronic supplementary material:**

The online version of this article (10.1186/s13287-018-0979-x) contains supplementary material, which is available to authorized users.

## Background

Primary biliary cholangitis (PBC) is a chronic cholestatic autoimmune liver disease, characterized by slow progressive destruction of small and medium-size intrahepatic bile ducts, which when untreated will culminate in end-stage biliary cirrhosis [1]. Various cell populations and cytokines are involved in PBC development, indicating a complex mechanism responsible for the pathogenesis of PBC [2, 3]. Currently, ursodeoxycholic acid (UDCA) and obeticholic acid (OCA) are approved by the US Food and Drug Administration to treat PBC with demonstrated clinical benefits [4–6]. However, about 25% to 40% of patients with PBC did not achieve a complete treatment response [7, 8]. Thus, it is necessary to explore new therapies for PBC.

Mesenchymal stem cells (MSCs) can be isolated from various tissues, including bone marrow, umbilical cord (UC), and adipose tissues [9]. Capable of differentiating into mesodermal cell lineages, MSCs also have a wide range of immunomodulatory properties [10]. In our previous studies, MSC transplantation (MSCT) showed therapeutic effects on various autoimmune diseases, including systemic lupus erythematosus [11, 12], primary Sjögren’s syndrome [13], and rheumatoid arthritis [14, 15]. In 2011, our early experiment in mice raised the possibility of applying syngeneic MSCs to treat PBC [16]. Subsequently, two other clinical trials reported that MSCT was feasible and well tolerated, thus providing an important clue for the treatment of patients with refractory PBC in the near future [17, 18]. However, very little is known regarding the underlying mechanism of the therapeutic effects of MSCT in PBC.

Currently, the mechanisms underlying the anti-inflammatory effects of MSCs are being investigated to establish the basis for the effective clinical application of MSCs. MSCs can express a high level of therapeutic factors to exert enhanced immunosuppressive function after interacting with a disease-specific environment [19–21]. Even when MSCs do not migrate directly to inflammation sites or injured tissues, they can still exert anti-inflammatory actions through secretory factors [22–25], such as galectins. Galectins are a group of galactoside-binding lectins that regulate various biological processes. Among these mammalian members, galectin-9 (Gal-9) is a 36-kDa tandem-repeat galectin [26, 27]. Treatment with recombinant Gal-9 ameliorates disease activity in various preclinical models of autoimmunity and allograft graft rejection [28–30]. However, whether UC-MSCs can produce Gal-9 to play an immunoregulatory role is not clear.

The aim of this study was to assess the efficacy of UC-MSCT in 2-octynoic acid coupled to bovine serum albumin (2OA-BSA)–induced murine autoimmune cholangitis and explore its underlying mechanisms. Here, we found that UC-MSC suppressed aberrant Th1 and Th17 responses via Gal-9 and repressed systemic and local inflammation in this PBC mouse model. Our results suggest that UC-MSCT may serve as an excellent therapeutic strategy for treating PBC.

## Methods

### Mice

Female C57BL/6 mice (6–8 weeks old) were obtained from the Laboratory Animal Center of the Affiliated Drum Tower Hospital of Nanjing University Medical School (Nanjing, Jiangsu, China). Mice were housed in a temperature-controlled environment with a 12 h light-dark cycle and were given standard laboratory chow and water *ad libitum*. All animal experiments were performed in accordance with the institutionally approved protocol for the use of animal research.

### Isolation of UC-MSC and cell culture

Fresh human UCs were obtained from the Affiliated Drum Tower Hospital of Nanjing University Medical School. UC-MSCs were prepared as described previously [31]. This study was approved by the ethics committee of the Affiliated Drum Tower Hospital of Nanjing University Medical School. To co-culture UC-MSCs with human biliary epithelial cells (BECs) (Jennio Biotech, Guangzhou, Guangdong, China), confluent BECs were added to the lower transwell chamber in 12-well plates treated with RPMI 1640 medium (Gibco, Life Technologies, Grand Island, NY, USA) supplemented with 10% fetal bovine serum (FBS) and 100 U/mL penicillin/streptomycin solution. Then UC-MSCs were seeded into the upper transwell chambers (Corning, New York, NY, USA) for 48 h. Interferon-gamma (IFN-γ) (20 ng/mL, PeproTech, Rocky Hill, NJ, USA) and α-lactose (10.8 mg/mL, Sigma-Aldrich, St. Louis, MO, USA) were added for the treatment of BECs before RNA extraction. All cells were maintained in an atmosphere with 95% humidity and 5% carbon dioxide at 37 °C.

### Induction of cholangitis and UC-MSCT

2OA (a synthetic chemical mimic of lipoic acid-lysine located within the inner domain of PDC-E2) was coupled to bovine serum albumin (2OA-BSA) as previously described [32]. For the induction of autoimmune cholangitis, 100 μg 2OA-BSA conjugate (in 50 μL phosphate-buffered saline, or PBS) was emulsified with 50 μL of complete Freund’s adjuvant (containing 1 mg/mL of mycobacterium tuberculosis strain H37RA, Sigma-Aldrich) and injected intraperitoneally (I.P.) into female C57BL/6 mice. Additionally, mice received 100 ng of pertussis toxin (List Biological Laboratories, Campbell, CA, USA) in 100 μL PBS I.P. at the time of initial immunization with 2OA-BSA and 2 days afterwards, respectively. Two weeks later, the mice were re-boosted I.P. with 100 μg 2OA-BSA in 50 μL PBS emulsified with 50 μL of incomplete Freund’s adjuvant (Sigma-Aldrich). The treatment of UC-MSCT in 2OA-BSA–immunized mice was performed after the onset of disease (that is, 8 weeks after immunization). Mice were injected intravenously with 1 × 10^6^ UC-MSCs and sacrificed 12 weeks after immunization.

### Serum biochemical markers, anti-PDC-E2 antibody, and cytokine measurement

The serum levels of alanine aminotransferase (ALT), aspartate aminotransferase (AST), alkaline phosphatase (ALP), and glutamyl transpeptidase (GGT) were determined by using detection kits (Jiancheng Bioengineering Institute, Nanjing, Jiangsu, China). The serum anti-PDC-E2 autoantibodies (LifeSpan Biosciences, Seattle, WA, USA) and human Gal-9 (RayBiotech, Norcross, GA, USA) in UC-MSC conditioned media (CM) were measured by enzyme-linked immunosorbent assay (ELISA) kits. Serum levels of IFN-γ, interleukin-17A (IL-17A), IL-12, and IL-23 were measured by using Milliplex Cytokine kits with Luminex (Millipore, Billerica, MA, USA). All procedures were performed in accordance with the instructions of the manufacturer.

### Histological analysis

Murine livers were harvested immediately after sacrifice, and aliquots were fixed in 10% buffered formalin at room temperature for 2 days, embedded in paraffin, and cut into 4 μm sections for routine hematoxylin (Dako Cytomation, Carpinteria, CA, USA) and eosin (American Master Tech Scientific, Lodi, CA, USA) (H&E) staining. Standard pathologic evaluation by light microscopy was performed for H&E-stained sections, and the relative levels of liver inflammation and biliary cell damage were recorded. Each section was scored as 0 = no pathologic change, 1 = minimal, 2 = mild, 3 = moderate, or 4 = severe in accordance with a widely accepted scoring system [33].

### Cell preparation and flow cytometry analysis

Livers, spleens, and peripheral blood were harvested immediately following sacrifice of the mice. Livers were first perfused with PBS containing 0.2% BSA (0.2% BSA/PBS), passed through a 100 μm nylon cell strainer, and re-suspended in 0.2% BSA/PBS. Hepatocytes were removed as pellets after centrifugation at 75*g* for 1 min, and the remaining suspended cells were collected. Spleens were disrupted between two glass slides and suspended in 0.2% BSA/PBS. Mononuclear cells from the livers were isolated by gradient centrifugation using 40% and 70% Percoll (Sigma-Aldrich). Peripheral blood mononuclear cells were obtained by lysis of erythrocytes in the blood. The following antibodies were used: anti-CD4, anti-IL-17A, and anti-IFN-γ (eBioscience, San Diego, CA, USA). For intracellular cytokine staining, cells were stimulated with 20 ng/mL phorbol-12-myristate-13-acetate plus 1 μg/mL ionomycin at 37 °C for 4–5 h in the presence of 5 μg/mL brefeldin A (all from Enzo Life Science, Farmingdale, NY, USA). Then the cells were fixed and permeabilized with a fixation/permeabilization kit (Nordic-MUbio, Maastricht, Limburg, the Netherlands), followed by staining with anti-IFN-γ or anti-IL-17A. Data were acquired by a FACS Calibur flow cytometer (BD Biosciences, Mountain View, CA, USA) and were analyzed with FlowJo software (Tree Star, Ashland, OR, USA).

### Cell labeling with GFP

To track the transplanted cells in vivo, UC-MSCs were labeled with green fluorescent protein (GFP) by lentivirus infection. Briefly, the pLV-CMV-GFP-Neo vector, PMD2.G and PSPAX2 packaging plasmids, and the X-treme GENE HP DNA Transfection Reagent (Roche, Basel, Switzerland) were added to 10% FBS Dulbecco’s Modified Eagle Medium, mixed gently, and incubated at room temperature for 20 min. The mixture was added dropwise into 293 T cells in a 10-cm plate. After 48 h of incubation, the virus supernatant was collected and filtered by using a 0.45-mm filter. UC-MSCs were then infected with the virus. After being co-cultured for 48 h, the aminoglycoside antibiotic G418 (Gibco-BRL, Carlsbad, CA, USA) was added to the medium at a final concentration of 600 mg/mL to select UC-MSCs with a stable GFP expression. The UC-MSCs labeled with GFP were observed with a fluorescence emission ratio at 530 nm by using an epifluorescence microscope and an excitation wavelength of 488 nm.

### Immunofluorescence

For the immunofluorescence examination, liver tissues were OCT-embedded, snap-frozen, and cut into 3 μm slides. Then slides were incubated with primary antibody overnight at 4 °C. Primary antibodies were as follows: Gal-9 (1:200; abcam, Cambridge, MA, USA). Slides were stained with 4′,6-diamidino-2-phenylindole (DAPI) and then examined by fluorescence microscope. The scale bar of the picture at 200× magnification represents 50 μm.

### Isolation and culture of naïve CD4^+^ T cells

Differentiation of murine Th1 and Th17 cells was performed as previously described [34, 35]. Briefly, for Th1 cell differentiation, naïve CD4^+^ T cells were purified from spleens by using a naïve CD4^+^ T cell Isolation Kit (Stem Cell Technologies, Vancouver, BC, Canada) and were cultured in 2.5 μg/mL anti-CD3 and 5 μg/mL anti-CD28 (eBioscience) pre-coated culture plates with 20 ng/mL IL-2 (PeproTech), 20 ng/mL IL-12 (PeproTech), and 5 μg/mL anti-IL-4 (eBioscience) for stimulation. For Th17 cell differentiation, naïve CD4^+^ T cells received the same treatment as for Th1 differentiation except 20 ng/mL IL-6 (PeproTech), 3 ng/mL transforming growth factor-beta (TGF-β) (PeproTech), 20 ng/mL IL-23 (PeproTech), 5 μg/mL anti-IFN-γ (eBioscience), and 5 μg/mL anti-IL-4 (eBioscience) were used for stimulation instead. To confirm the role of Gal-9 in UC-MSCs, the cells were treated with UC-MSCs alone or UC-MSCs plus 10.8 mg/mL α-lactose (Sigma-Aldrich). After culture for 72 h, the cells were collected for further analysis.

### Proliferation assay

Murine CD4^+^ T cells were purified from spleens in accordance with the instructions of the manufacturer (BD Biosciences). For the proliferation assay, CD4^+^ T cells at a density of 1 × 10^6^ cells per well were labeled with 5 μM carboxyfluorescein diacetate succinimidyl ester (CFSE) (Invitrogen, Camarillo, CA, USA) and then co-cultured with UC-MSCs (1 × 10^5^ cells per well) or UC-MSCs with 10.8 mg/mL α-lactose (Sigma-Aldrich) for 4 days. Cell division was determined by measuring the CFSE fluorescence intensity by flow cytometry.

### RNA isolation and real-time polymerase chain reaction

Total RNA was extracted from liver tissues, UC-MSCs, and BECs by using TRIzol (Vazyme Biotech, Nanjing, Jiangsu, China) and reverse-transcribed by PrimeScript™ RT Master Mix (Vazyme Biotech). Quantitative real-time polymerase chain reaction (PCR) assays using gene-specific primers (Table 1) and SYBR Premix Ex Taq kit (Vazyme Biotech) were run on StepOnePlus™ Real Time PCR Systems (Applied Biosystems, Foster City, CA, USA). The relative expression of each gene was determined and normalized to the expression of glyceraldehyde 3-phosphate dehydrogenase (*GAPDH*). Relative quantification was calculated by using the 2^–∆∆Ct^ method.

**Table 1:** Primers for real-time PCR

Genes	Primers	
	Forward (5‘-3’)	Reverse (5‘-3’)
Human GAPDH	GCACCGTCAAGGCTGAGAAC	TGGTGAAGACGCCAGTGGA
Human Galectin-1	AGGTTGTTGCTGTCTTTGCC	CAAACCTGGAGAGTGCCTTC
Human Galectin-3	TGCAACCTTGAAGTGGTCAG	GGGGAAGGGAAGAAAGACAG
Human Galectin-9	ATAGTCCCAGAAAAGGGGACA	TCCTAGTGGGTGTGAAAGGC
Human CXCL10	GCTGATGCAGGTACAGCGT	CACCATGAATCAAACTGCGA
Human CCL5	TGTACTCCCGAACCCATTTC	TACACCAGTGGCAAGTGCTC
Human CCL20	CGTGTGAAGCCCACAATAAA	GTGCTGCTACTCCACCTCTG
Human CX3CL1	ACGTGATGTTGCATTTCGTC	CCGATATCTCTGTCGTGGCT
Mouse GAPDH	TTGATGGCAACAATCTCCAC	CGTCCCGTAGACAAAATGGT
Mouse IFN-γ	ATGAACGCTACACACTGCATC	CCATCCTTTTGCCAGTTCCTC
Mouse IL-17A	TTTAACTCCCTTGGCGCAAAA	CTTTCCCTCCGCATTGACAC
Mouse IL-12	GCTTCTCCCACAGGAGGTTT	CTAGACAAGGGCATGCTGGT
Mouse IL-23	GCTCCCCTTTGAAGATGTCA	GACCCACAAGGACTCAAGGA
Mouse T-bet	ATCCTGTAATGGCTTGTGGG	TCAACCAGCACCAGACAGAG
Mouse RORγt	GGTGATAACCCCGTAGTGGA	CTGCAAAGAAGACCCACACC
Mouse CXCL10	CCTATGGCCCTCATTCTCAC	CTCATCCTGCTGGGTCTGAG
Mouse CCL5	CCACTTCTTCTCTGGGTTGG	GTGCCCACGTCAAGGAGTAT
Mouse CCL20	TGTACGAGAGGCAACAGTCG	TCTGCTCTTCCTTGCTTTGG
Mouse CX3CL1	TGGGATTCGTGAGGTCATCT	CGCGTTCTTCCATTTGTGTA

### Western blot assay

For Western blot assay, rabbit anti-Gal-9 antibody (Proteintech, Wuhan, Hubei, China), rabbit antibodies to the kinases c-Jun N-terminal kinase (JNK), phospho-JNK, p38/mitogen-activated protein kinase (p38/MAPK), phospho-p38/MAPK, extracellular-regulated protein kinase (ERK), phospho-ERK, signal transducer and activator of transcription 1 (STAT1), phospho-STAT1, STAT3, phospho-STAT3, STAT5, phospho-STAT5 (Cell Signaling Technology (CST), Danvers, MA, USA), and mouse anti-GAPDH antibody (Proteintech) were used as primary antibodies. Horseradish peroxidase (HRP)-conjugated anti-rabbit IgG (H + L) and anti-mouse IgG (H + L) (Proteintech) were used as secondary antibodies, respectively. UC-MSCs were washed twice with PBS and lysed on ice for 30 min with 1× radioimmunoprecipitation assay (RIPA) buffer (CST) containing 1% 100× protease/phosphatase inhibitor cocktail (CST). Lysates were centrifuged at 12,000*g* at 4 °C for 20 min, and the supernatants were subjected to sodium dodecyl sulfate-polyacrylamide gel electrophoresis (SDS-PAGE). Protein was transferred to polyvinylidene fluoride membranes (Millipore), blocked for 1 h in 5% nonfat milk in TBST (10 mM Tris (BioSharp, Hefei, Anhui, China) (pH 7) and 150 mM NaCl, 0.1% Tween 20), and immunoblotted with the above-listed primary antibodies and appropriate HRP-conjugated secondary antibodies. Chemiluminescence HRP substrate (Millipore) was used to detect the specific proteins, and the bands were visualized by using the G:BOX gel imaging system (Syngene, Cambridge, UK). Analysis was performed by using ImageJ software (National Institutes of Health, Bethesda, MD, USA).

### Statistical analysis

Data are presented as the mean ± standard error of the mean (SEM). Student’s *t* test was applied when two groups were compared for significant differences. A one-way analysis of variance (ANOVA) followed by the Newman–Keuls test was used to compare more than two groups. The Prism statistical package (GraphPad Software Inc., La Jolla, CA, USA) was used. A *P* value of less than 0.05 was considered statistically significant.

## Results

### UC-MSCT alleviates 2OA-BSA–induced autoimmune cholangitis

**Fig. 1:**
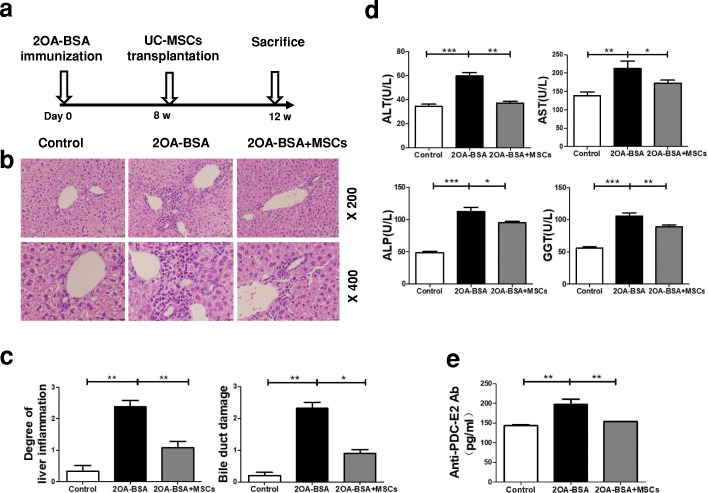
Umbilical cord–derived mesenchymal stem cell transplantation alleviates 2-octynoic acid coupled to bovine serum albumin (2OA-BSA)–induced autoimmune cholangitis. **a** Schematic illustration of the experimental procedure. **b** Representative images of liver tissue sections by hematoxylin-and-eosin staining from control, primary biliary cholangitis, or umbilical cord–derived mesenchymal stem cell (UC-MSC)–treated mice, showing liver inflammation and the extent of bile duct damage. **c** Histological scores were assessed on the basis of lymphocytic infiltration and bile duct damage in the liver. **d** The serum levels of alanine aminotransferase (ALT), aspartate aminotransferase (AST), alkaline phosphatase (ALP), and glutamyl transpeptidase (GGT) were measured. **e** The levels of anti-PDC-E2 autoantibodies were measured. Bars represent the mean ± standard error of the mean (SEM); *n* = 6 per group. *P <0.05, **P <0.01, ***P <0.001

To examine the potential therapeutic effect of UC-MSCs in PBC, we used the standard protocol to induce cholangitis in mice by 2OA-BSA immunization (Fig. 1a). As shown in Fig. 1b and c, intravenous UC-MSCT significantly mitigated the severity of liver inflammation and bile duct damage by histological analysis. The amelioration was further confirmed by reduced levels of ALT, AST, ALP, and GGT as compared with untreated mice (Fig. 1d). More importantly, anti-PDC-E2 autoantibodies in the serum were significantly decreased after UC-MSCT (Fig. 1e). These results indicated that UC-MSCT effectively suppressed disease development in the experimental autoimmune cholangitis mice.

### UC-MSCT inhibits the aberrant Th1 and Th17 responses in 2OA-BSA–induced autoimmune cholangitis

**Fig. 2:**
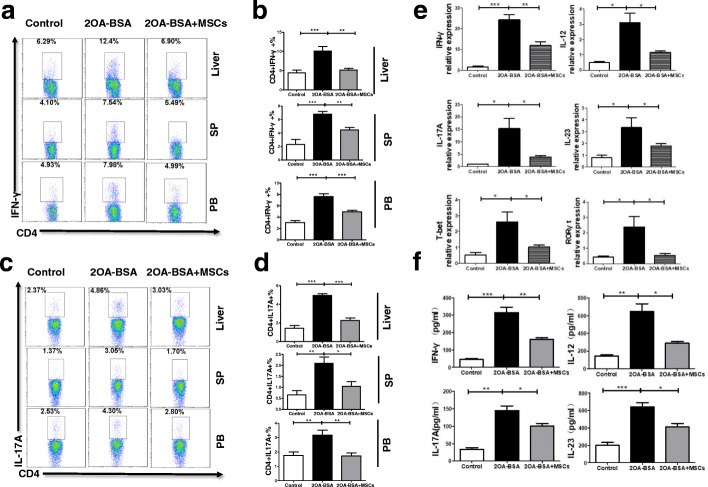
Umbilical cord–derived mesenchymal stem cell transplantation inhibits the aberrant T helper 1 (Th1) and Th17 responses in 2-octynoic acid coupled to bovine serum albumin (2OA-BSA)–induced autoimmune cholangitis. **a** Representative flow cytometric profiles showing the frequencies of Th1 cells in the liver, spleen, and blood. **b** The frequencies of Th1 cells were quantified. **c** Representative flow cytometric profiles showing the frequencies of Th17 cells in the liver, spleen, and blood. **d** The frequencies of Th17 cells were quantified. **e** Intrahepatic mRNA expressions of interferon-gamma (IFN-γ), interleukin-17A (IL-17A), IL-12, IL-23, T-bet, and RAR-related orphan receptor gamma t (RORγt) were determined by real-time polymerase chain reaction. **f** Levels of IFN-γ, IL-17A, IL-12, and IL-23 in serum were detected by Luminex. Bars represent the mean ± standard error of the mean (SEM); *n* = 6 per group. *P <0.05, **P <0.01, ***P <0.001. Abbreviations: *PB* peripheral blood, *SP* spleen

As shown in Fig. 2a–d, UC-MSCT significantly decreased the infiltration of Th1 and Th17 cells in the liver, spleen, and peripheral blood in 2OA-BSA–immunized mice. Although the frequencies of Th2 cells did not change, UC-MSCT remarkably downregulated the Th1/Th2 ratio (Additional file 1: Figures S1 and S2). Besides, we confirmed that the infusion of UC-MSCs into normal C57BL/6 mice did not induce cross-species immunoreaction (Additional file 1: Figure S3). We then analyzed the mRNA expressions of IFN-γ, IL-12, IL-17A, and IL-23 in liver and found that these cytokines were downregulated in the UC-MSCT group. In addition, UC-MSCT suppressed the mRNA expression of T-bet and RAR-related orphan receptor gamma t (RORγt), the most important transcription factors for Th1 and Th17 cells, respectively (Fig. 2e). Consistently, quantification of IFN-γ, IL-12, IL-17A, and IL-23 expression revealed that UC-MSCT significantly decreased their levels in the serum (Fig. 2f). Therefore, the above data indicated that UC-MSCT resulted in attenuation of the Th1 and Th17 immune responses in 2OA-BSA–immunized mice.

### Gal-9 is highly expressed in UC-MSCs

IFN-γ was reported to be overexpressed in the serum and liver of patients with PBC [36]. Although inflammatory factors affect the immunomodulatory effects of MSCs [37], it is still unknown whether this immunoregulatory activity could be affected by IFN-γ in the context of PBC. Prostaglandin E2 (PGE2) [22], nitric oxide [23], and indoleamine-pyrrole 2,3-dioxygenase (IDO) [24] have been demonstrated to be involved in the functions of MSCs. Nevertheless, blockage of any one of these molecules is insufficient to completely abolish the immunoregulatory effects of MSCs, indicating that some other important mediators may also play a role. Previous studies have found that human bone marrow–derived MSCs (BM-MSCs) could secrete Gal-1 or Gal-3 to exert immunomodulatory functions [38, 39]. However, whether the Galectin family member acts as a major effector in the process of UC-MSC immunomodulation is still to be elucidated.

**Fig. 3:**
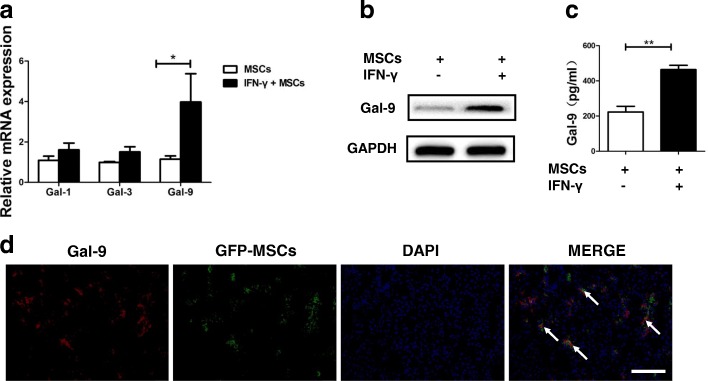
Galectin-9 (Gal-9) is highly expressed in umbilical cord–derived mesenchymal stem cells (UC-MSCs). **a** Total mRNA of UC-MSCs was extracted after activation with or without interferon-gamma (IFN-γ) and analyzed by real-time polymerase chain reaction for the expression of Gal-1, Gal-3, and Gal-9. Bars represent the mean ± standard error of the mean (SEM); *n* = 4 per group. **b**, **c** UC-MSCs were treated with or without IFN-γ and analyzed by Western blot and enzyme-linked immunosorbent assay for the expression of Gal-9. Bars represent the mean ± SEM; *n* = 3 per group. **d** The fluorescence microscope was employed to detect green fluorescent protein (GFP)-positive UC-MSCs in the livers of primary biliary cholangitis mice. Colocalization (white arrow) of the GFP (green) with Gal-9 (red) staining indicated that UC-MSCs expressed Gal-9. Scale bar = 50 μm. Abbreviation: *DAPI* 4′,6-diamidino-2-phenylindole

We then attempted to investigate the expression of Gal-1, Gal-3, and Gal-9 in the UC-MSCs after IFN-γ stimulation. The results showed that only Gal-9 significantly increased after IFN-γ stimulation (Fig. 3a), further confirmed by the ELISA and Western blot assay (Fig. 3b, c). Meanwhile, we detected that a portion of UC-MSCs were localized in the liver, which indicated that UC-MSCs could migrate to the liver injury site. Moreover, double-positive yellow cells with a stretching morphology were observed in UC-MSC–infused livers, which suggested that UC-MSCs could express Gal-9 efficiency in vivo (Fig. 3d). Intriguingly, the concentration of Gal-9 was shown to significantly increase in the UC-MSC–treated PBC mouse model, in both the serum and livers (Additional file 1: Figure S4).

### Via Gal-9, UC-MSCs suppress CD4^+^ T-cell proliferation as well as Th1 and Th17 cell differentiation

**Fig. 4:**
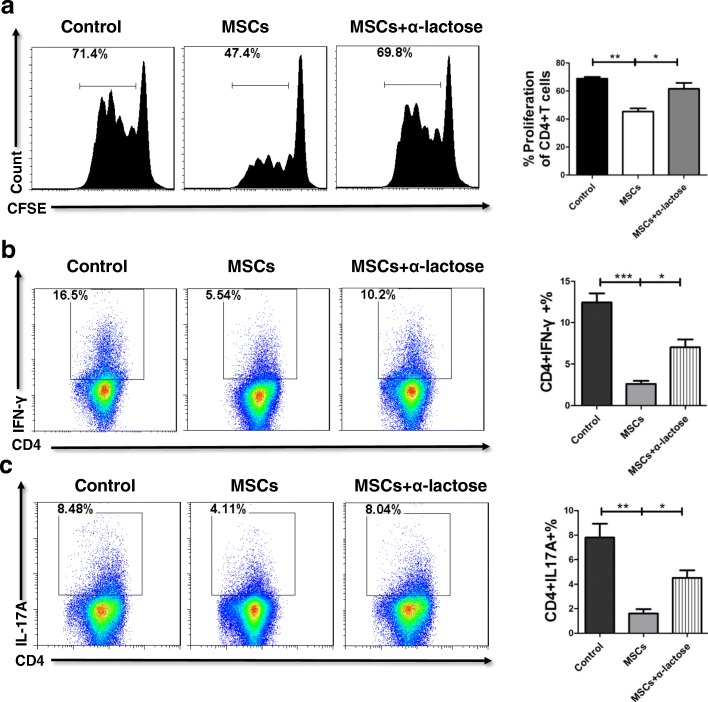
Umbilical cord–derived mesenchymal stem cells (UC-MSCs) suppress CD4^+^ T-cell proliferation as well as T helper 1 (Th1) and Th17 cell differentiation via galectin-9. **a** Carboxyfluorescein diacetate succinimidyl ester (CFSE)–labeled CD4^+^ T cells were cultured in the presence of UC-MSCs or UC-MSCs plus α-lactose for 4 days. The proliferation of murine CD4^+^ T cells was assessed on the basis of the fluorescence intensity of CFSE. Bars represent the mean ± standard error of the mean (SEM); *n* = 3 per group. **b** Purified naïve murine CD4^+^ T cells were cultured under Th1 polarization conditions in the presence of UC-MSCs or UC-MSCs plus α-lactose. Representative flow cytometric profiles that show the frequencies of Th1 cells are presented. The frequencies of Th1 cells were quantified. **c** Purified naïve murine CD4^+^ T cells were cultured under Th17 polarization conditions in the presence of UC-MSCs or UC-MSCs plus α-lactose. Representative flow cytometric profiles that show the frequencies of Th17 cells are presented. The frequencies of Th17 cells were quantified. Bars represent the mean ± SEM; *n* = 4 per group. *P <0.05, **P <0.01, ***P <0.001

Next, we aimed to explore whether Gal-9 contributed to the immunomodulatory properties of UC-MSCs. UC-MSCs significantly inhibited the proliferation of CD4^+^ T cells by decreasing about 30%; in contrast, this effect was impaired after adding α-lactose, a natural inhibitor of Gal-9 (Fig. 4a). Furthermore, the differentiation of Th1 and Th17 cells was inhibited in the presence of UC-MSCs from 16.5% to 5.54% and 8.48% to 4.11%, respectively. However, upon the addition of α-lactose, the proportions of Th1 and Th17 cells reversely increased about 50% (Fig. 4b, c). Since UC-MSCs secrete the soluble form of Gal-9, we treated the cells with CM collected from UC-MSCs. We found that MSC-CM also significantly inhibited CD4^+^ cell proliferation (Additional file 1: Figure S5a) and downregulated the percentage of Th1 and Th17 cells; moreover, the addition of α-lactose blocked the inhibitory effects of MSC-CM (Additional file 1: Figure S5b, c). Taken together, these results provided evidence that Gal-9 participated in the immunoregulatory functions of UC-MSCs by mediating the proliferation and differentiation of T-cell populations.

### UC-MSCT downregulates proinflammatory chemokines to repress inflammatory responses

**Fig. 5:**
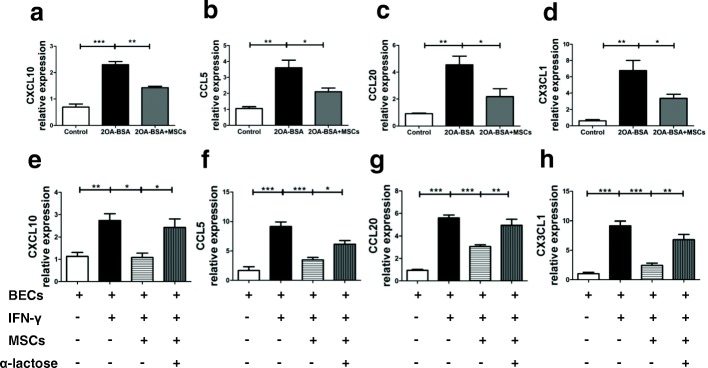
Umbilical cord–derived mesenchymal stem cell (UC-MSC) transplantation downregulates proinflammatory chemokines to repress inflammatory responses. **a**–**d** Intrahepatic mRNA expressions of CCL5, CCL20, CXCL10, and CX3CL1 were determined by real-time polymerase chain reaction. Bars represent the mean ± standard error of the mean (SEM); *n* = 6 per group. **e**–**h** CCL5, CCL20, CXCL10, and CX3CL1 mRNA expression in unstimulated, interferon-gamma (IFN-γ)–stimulated, UC-MSCs or UC-MSCs plus α-lactose co-cultured with biliary epithelial cells (BECs) demonstrated by real-time polymerase chain reaction. Bars represent the mean ± SEM; *n* = 4 per group. *P <0.05, **P <0.01, ***P <0.001. Abbreviation: *2OA-BSA* 2-octynoic acid coupled to bovine serum albumin

Chemokines direct migration and localization of lymphocytes within tissues, thus playing important roles in regulating inflammatory responses [40]. To ascertain whether UC-MSCs affect the expression of chemokines, we further measured the hepatic mRNA expression of several proinflammatory chemokines in vivo. As shown in Fig. 5a–d, UC-MSCT significantly downregulated the intrahepatic mRNA expressions of CCL5, CCL20, CXCL10, and CX3CL1. Notably, the expression of CXC3CL1 and CCL20 decreased by about 50% after UC-MSCT. To further evaluate whether UC-MSCs protected the BECs through Gal-9, we co-cultured UC-MSCs with BECs before IFN-γ stimulation in vitro to examine the alteration of these chemokines. Expression of CCL5, CCL20, CXCL10, and CX3CL1 mRNA was significantly reduced in BECs after UC-MSC treatment, while this cytoprotective function of UC-MSCs was impaired after the addition of α-lactose (Fig. 5e–h). Thus, these data implied that inflammatory cytokines stimulated chemokine expression by BECs, leading to the recruitment of Th1 and Th17 cells to the bile ducts. UC-MSCs could reduce these proinflammatory chemokines to suppress aberrant lymphocytic infiltration with Gal-9 participating in this process.

### Upregulation of Gal-9 in UC-MSCs induced by IFN-γ is mediated through JNK and STAT signaling pathways

**Fig. 6:**
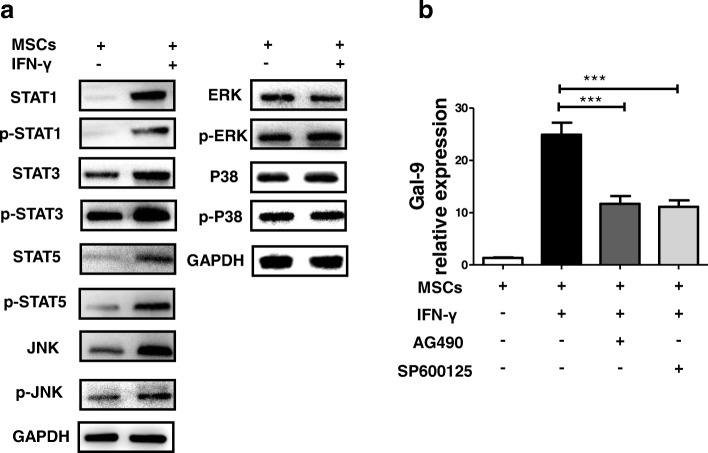
Interferon-gamma (IFN-γ)–induced galectin-9 (Gal-9) upregulation of umbilical cord–derived mesenchymal stem cells (UC-MSCs) is mediated through the c-Jun N-terminal kinase (JNK) and signal transducer and activator of transcription (STAT) signaling pathways. **a** The expression of STAT, p38/mitogen-activated protein kinase (p38/MAPK), extracellular-regulated protein kinase (ERK), JNK, and their phosphorylation forms were detected by Western blot analysis in unstimulated and IFN-γ–stimulated UC-MSCs. **b** Total mRNA of UC-MSCs was extracted after the activation of IFN-γ with JNK or STAT inhibitors and then analyzed by real-time polymerase chain reaction for the expression of Gal-9. Bars represent the mean ± standard error of the mean (SEM); *n* = 3 per group. ***P <0.001

To elucidate the possible signaling pathways involved in IFN-γ–induced Gal-9 expression, various signaling pathways were investigated. The administration of IFN-γ effectively activated the STATs (STAT1, STAT3, and STAT5) and JNK signaling pathways in UC-MSCs, whereas the expression of p38/MAPK and ERK was not significantly changed by Western blot (Fig. 6a). In an effort to verify these results, UC-MSCs were treated with SP600125 (JNK inhibitor) or AG490 (STAT inhibitor) upon IFN-γ stimulation and were assessed to detect Gal-9 expression. As shown in Fig. 6b, we found that SP600125 and AG490 significantly suppressed the production of Gal-9 in UC-MSCs, indicating that the induction of Gal-9 was likely to be regulated by the STAT and JNK signaling pathways.

## Discussion

In the present study, we demonstrated that UC-MSCT was able to attenuate the development of 2OA-BSA–induced autoimmune cholangitis by inhibiting immune response mediated by Th1/Th17 cells and downregulating chemokine activities. Furthermore, our data also indicated that the regulation of CD4^+^ T-cell proliferation, the differentiation of Th1 and Th17 cells, and the cytoprotection by UC-MSCs were dependent on Gal-9, the production of which involved the STAT and JNK signaling pathways.

PBC is an autoimmune disease associated with organ-specific and systemic autoimmunity. Autoreactive CD4^+^ T cells have been linked to the pathogenesis of PBC. Th1 cells with their signature cytokine IFN-γ as well as Th17 cells with IL-17A are thought to be the main pathogenic mediators in PBC [41]. Here, we used a well-recognized murine model of autoimmune cholangitis to investigate the potential therapeutic effects of UC-MSCT and its underlying mechanism. In this study, on one hand, UC-MSCT decreased the frequencies of Th1 and Th17 cells in both systemic and local tissues in 2OA-BSA–immunized mice; on the other hand, UC-MSCT suppressed the expression of inflammatory chemokines. Previous studies have reported that upregulation of regulatory T (Treg) cells is one important aspect of MSC-mediated immunomodulation [42]. Accordingly, we also examined the alteration of Treg cells and found that the frequency of Treg cells in the peripheral blood was elevated after UC-MSCT, which suggested that the diminished Th1 and Th17 cell responses in the peripheral blood were due in part to the expansion of Treg cells (Additional file 1: Figure S6).

It is well known that MSCs require proinflammatory cytokines to induce their immunosuppressive function [43]. Galectins, a family of beta-galactoside–binding proteins, have emerged as one of the main regulators of the MSC immunosuppressive function [44]. Gal-9 is a unique member of this family and has a wide variety of biological functions in innate and adaptive immunity [45, 46]. Gal-9 was strongly induced in human BM-MSCs [47]. Ungerer et al. demonstrated that Gal-9 was a major mediator of the anti-proliferative effects of BM-MSCs [48]. Here, we found that Gal-9 was constitutively expressed in UC-MSCs and that IFN-γ stimulation could induce substantially higher Gal-9 expression in UC-MSCs. Of interest, we found that the amount of IFN-γ–positive cells was widely distributed around interlobular bile ducts of the PBC mouse model (Additional file 1: Figure S7) and the expression of Gal-9 was elevated after UC-MSCT, further supporting the concept that the disease-specific microenvironment is crucial for the therapeutic effects of implanted MSCs.

According to current knowledge, MSCs may exert their therapeutic effects in two ways: by cell replacement or in a paracrine manner. The relative importance of these two mechanisms is still a topic of controversy [49–51]. In the present study, it was noticed that the number of transplanted UC-MSCs decreased gradually with time but that the expression of Gal-9 was significantly increased (Additional file 1: Figure S8). Considering the histological and serological improvement by UC-MSCT lasted for a long term while only a small portion of UC-MSCs were observed in the liver within 7 days after infusion, we speculated that the beneficial effects of UC-MSCs on the recovery of liver injury may be attributed to their secretome of soluble factors, such as Gal-9. Mechanistically, the results of our in vitro studies indicated that UC-MSC–derived Gal-9 could suppress the proliferation and differentiation of CD4^+^ T cells and thereby alleviate experimental autoimmune cholangitis.

Chemokines represent one kind of major mediators of innate immunity as they play a key role in the selective recruitment of immune cells during localized inflammatory responses [52]. The potential contribution of chemokines and inflammation to the progression of PBC in the chemokine-chemokine receptor network may provide important clues in the injury of BECs in PBC [53–55]. Our data showed that UC-MSCT decreased the expression of these proinflammatory chemokines; however, this cytoprotective function was impaired after the inhibition of Gal-9, which suggests that UC-MSCs could protect damaged BECs in a paracrine manner. Based on these findings, we suppose that BEC-induced chemokines are likely to be active players in PBC pathogenesis and may elicit the migration and infiltration of autoreactive T cells, which further lead to the liver lesions observed in PBC. Moreover, our results emphasize the potential of the downregulation of specific chemokines as one of the effective ways to modulate inflammatory responses during UC-MSCT.

STAT, p38/MAPK, ERK, and JNK can be activated by IFN-γ [56]. These pathways regulate the expression of genes involved in the initiation, propagation, and resolution of immune and inflammatory responses. Thus, the focus of this study was to explore whether the IFN-γ–induced expression of Gal-9 in UC-MSCs depends upon these kinase pathways. We demonstrated that treatment with STAT and JNK inhibitors effectively repressed Gal-9 expression induced by IFN-γ in UC-MSCs. To the best of our knowledge, this is the first study to demonstrate that the induction of Gal-9 in UC-MSCs is mediated by the activation of the STAT and JNK signaling pathways.

## Conclusions

In summary, the present study shows that UC-MSCs exert profound inhibitory effects on inflammatory responses to alleviate liver injury in experimental autoimmune cholangitis mice. Furthermore, UC-MSCs inhibit Th1 and Th17 cell responses as well as aberrant chemokine activities through Gal-9–mediated immunosuppression. Additionally, the induction of Gal-9 in UC-MSCs is mediated by the STAT and JNK signaling pathways. Our results provide novel insights into the clinical application of UC-MSCs in the treatment of PBC.

## Additional files

Additional file 1: **Figure S1.** Umbilical cord–derived mesenchymal stem cell transplantation does not influence Th2 cells. *n* = 6; Bars represent the mean ± SEM; Abbreviations: *PB* peripheral blood, *SP* spleen. **Figure S2.** Umbilical cord–derived mesenchymal stem cell transplantation downregulates Th1/Th2 ratio. *n* = 6; Bars represent the mean ± SEM; *P <0.05, **P <0.01. Abbreviations: *PB* peripheral blood, *SP* spleen. **Figure S3.** Umbilical cord–derived mesenchymal stem cell transplantation does not affect the Th1 and Th17 immune responses in normal mice. (**a**, **b**) The alterations of Th1 and Th17 cells in different groups. *n* = 4; Bars represent the mean ± SEM; Abbreviations: *PB* peripheral blood, *SP* spleen. **Figure S4.** Umbilical cord–derived mesenchymal stem cell transplantation upregulates the expression of galectin-9 (Gal-9). **a** The serum levels of Gal-9 in different groups. *n* = 6. **b** The liver expression of Gal-9 in different groups. *n* = 3; Bars represent the mean ± SEM; **P <0.01, ***P <0.001. Abbreviation: *GAPDH* glyceraldehyde 3-phosphate dehydrogenase. **Figure S5.** Galectin-9(Gal-9) contributes to the immunoregulatory function of umbilical cord–derived mesenchymal stem cell conditioned media (MSC-CM). (**a**-**c**) The proliferation of CD4+ T cells, the alterations of Th1 and Th17 cells in different groups. *n* = 4; Bars represent the mean ± SEM; *P <0.05, **P <0.01, ***P <0.001. Abbreviations: *CFSE* Carboxyfluorescein diacetate succinimidyl ester. **Figure S6.** Umbilical cord–derived mesenchymal stem cell transplantation upregulates the regulatory T (Treg) cells. The alterations of Treg cells in different groups. *n* = 6; Bars represent the mean ± SEM; *P <0.05, **P <0.01, ***P <0.001. Abbreviations: *PB* peripheral blood, *SP* spleen. **Figure S7.** Specific staining of interferon-gamma (IFN-γ) in the liver section. Scale bar = 32 μm. **Figure S8.** Dynamic changes of engraftment umbilical cord–derived mesenchymal stem cell (UC-MSC) and galectin-9 (Gal-9) in the liver section. Scale bar = 50 μm. Abbreviation: *GFP*+ green fluorescent protein–positive, *Gal-9*+, galectin-9, *DAPI* 4′,6-diamidino-2-phenylindole. (DOCX 779 kb)

Additional file 2: Supplementary Materials and Methods. (DOCX 21 kb)

## Abbreviations

2OA-BSA: 2-octynoic acid coupled to bovine serum albumin
ALP: Alkaline phosphatase
ALT: Alanine aminotransferase
AST: Aspartate aminotransferase
BEC: Biliary epithelial cell
BM-MSC: Bone marrow–derived mesenchymal stem cell
CFSE: Carboxyfluorescein diacetate succinimidyl ester
CM: Conditioned media
CST: Cell Signaling Technology
ELISA: Enzyme-linked immunosorbent assay
ERK: Extracellular-regulated protein kinase
FBS: Fetal bovine serum
Gal-9: Galectin-9
GAPDH: Glyceraldehyde 3-phosphate dehydrogenase
GFP: Green fluorescent protein
GGT: Glutamyl transpeptidase
H&E: Hematoxylin and eosin
HRP: Horseradish peroxidase
IFN-γ: Interferon-gamma
IL: Interleukin
I.P.: Intraperitoneally
JNK: c-Jun N-terminal kinase
MSC: Mesenchymal stem cell
MSCT: Mesenchymal stem cell transplantation
p38/MAPK: p38/mitogen-activated protein kinase
PBC: Primary biliary cholangitis
PBS: Phosphate-buffered saline
PCR: Polymerase chain reaction
STAT: Signal transducer and activator of transcription
Treg: Regulatory T
UC: Umbilical cord
UC-MSC: Umbilical cord–derived mesenchymal stem cell
UC-MSCT: Umbilical cord–derived mesenchymal stem cell transplantation

## References

[1] Griffiths L, Dyson JK, Jones DE (2014). The new epidemiology of primary biliary cirrhosis. Semin Liver Dis..

[2] Mousa HS, Carbone M, Malinverno F, Ronca V, Gershwin ME, Invernizzi P (2016). Novel therapeutics for primary biliary cholangitis: toward a disease-stage-based approach. Autoimmun Rev..

[3] Hirschfield GM, Gershwin ME (2013). The immunobiology and pathophysiology of primary biliary cirrhosis. Annu Rev Pathol..

[4] Hirschfield GM, Mason A, Luketic V, Lindor K, Gordon SC, Mayo M (2015). Efficacy of obeticholic acid in patients with primary biliary cirrhosis and inadequate response to ursodeoxycholic acid. Gastroenterology.

[5] Markham A, Keam SJ (2016). Obeticholic acid: first global approval. Drugs.

[6] Nevens F, Andreone P, Mazzella G, Strasser SI, Bowlus C, Invernizzi P (2016). A Placebo-Controlled Trial of Obeticholic Acid in Primary Biliary Cholangitis. N Engl J Med.

[7] Spacek LA, Solga SF (2016). Obeticholic acid in primary biliary cholangitis. N Engl J Med..

[8] Chascsa D, Carey EJ, Lindor KD (2017). Old and new treatments for primary biliary cholangitis. Liver Int..

[9] Tyndall A (2014). Mesenchymal stem cell treatments in rheumatology: a glass half full?. Nat Rev Rheumatol..

[10] Shi Y, Hu G, Su J, Li W, Chen Q, Shou P (2010). Mesenchymal stem cells: a new strategy for immunosuppression and tissue repair. Cell Res..

[11] Liang J, Zhang H, Hua B, Wang H, Lu L, Shi S (2010). Allogenic mesenchymal stem cells transplantation in refractory systemic lupus erythematosus: a pilot clinical study. Ann Rheum Dis..

[12] Sun L, Wang D, Liang J, Zhang H, Feng X, Wang H (2010). Umbilical cord mesenchymal stem cell transplantation in severe and refractory systemic lupus erythematosus. Arthritis Rheum..

[13] Xu J, Wang D, Liu D, Fan Z, Zhang H, Liu O (2012). Allogeneic mesenchymal stem cell treatment alleviates experimental and clinical Sjogren syndrome. Blood.

[14] Liu R, Li X, Zhang Z, Zhou M, Sun Y, Su D (2015). Allogeneic mesenchymal stem cells inhibited T follicular helper cell generation in rheumatoid arthritis. Sci Rep..

[15] Sun Y, Kong W, Huang S, Shi B, Zhang H, Chen W (2017). Comparable therapeutic potential of umbilical cord mesenchymal stem cells in collagen-induced arthritis to TNF inhibitor or anti-CD20 treatment. Clin Exp Rheumatol..

[16] Wang D, Zhang H, Liang J, Gu Z, Ma X, Huang J (2011). Effect of allogeneic bone marrow-derived mesenchymal stem cells transplantation in a polyI:C-induced primary biliary cirrhosis mouse model. Clin Exp Med..

[17] Wang L, Han Q, Chen H, Wang K, Shan GL, Kong F (2014). Allogeneic bone marrow mesenchymal stem cell transplantation in patients with UDCA-resistant primary biliary cirrhosis. Stem Cells Dev..

[18] Wang L, Li J, Liu H, Li Y, Fu J, Sun Y (2013). Pilot study of umbilical cord-derived mesenchymal stem cell transfusion in patients with primary biliary cirrhosis. J Gastroenterol Hepatol.

[19] Krampera M, Cosmi L, Angeli R, Pasini A, Liotta F, Andreini A (2006). Role for interferon-gamma in the immunomodulatory activity of human bone marrow mesenchymal stem cells. Stem Cells.

[20] Ryan JM, Barry F, Murphy JM, Mahon BP (2007). Interferon-gamma does not break, but promotes the immunosuppressive capacity of adult human mesenchymal stem cells. Clin Exp Immunol..

[21] Liu Y, Wang L, Kikuiri T, Akiyama K, Chen C, Xu X (2011). Mesenchymal stem cell-based tissue regeneration is governed by recipient T lymphocytes via IFN-gamma and TNF-alpha. Nat Med..

[22] Nemeth K, Leelahavanichkul A, Yuen PS, Mayer B, Parmelee A, Doi K (2009). Bone marrow stromal cells attenuate sepsis via prostaglandin E(2)-dependent reprogramming of host macrophages to increase their interleukin-10 production. Nat Med..

[23] Sato K, Ozaki K, Oh I, Meguro A, Hatanaka K, Nagai T (2007). Nitric oxide plays a critical role in suppression of T-cell proliferation by mesenchymal stem cells. Blood.

[24] Ling W, Zhang J, Yuan Z, Ren G, Zhang L, Chen X (2014). Mesenchymal stem cells use IDO to regulate immunity in tumor microenvironment. Cancer Res..

[25] Xu C, Yu P, Han X, Du L, Gan J, Wang Y (2014). TGF-beta promotes immune responses in the presence of mesenchymal stem cells. J Immunol..

[26] Thijssen VL, Poirier F, Baum LG, Griffioen AW (2007). Galectins in the tumor endothelium: opportunities for combined cancer therapy. Blood.

[27] Grigorian A, Torossian S, Demetriou M (2009). T-cell growth, cell surface organization, and the galectin-glycoprotein lattice. Immunol Rev..

[28] Sakai K, Kawata E, Ashihara E, Nakagawa Y, Yamauchi A, Yao H (2011). Galectin-9 ameliorates acute GVH disease through the induction of T-cell apoptosis. Eur J Immunol..

[29] Hirao H, Uchida Y, Kadono K, Tanaka H, Niki T, Yamauchi A (2015). The protective function of galectin-9 in liver ischemia and reperfusion injury in mice. Liver Transpl..

[30] Seki M, Oomizu S, Sakata KM, Sakata A, Arikawa T, Watanabe K (2008). Galectin-9 suppresses the generation of Th17, promotes the induction of regulatory T cells, and regulates experimental autoimmune arthritis. Clin Immunol..

[31] Zhao C, Zhang L, Kong W, Liang J, Xu X, Wu H (2015). Umbilical cord-derived mesenchymal stem cells inhibit Cadherin-11 expression by fibroblast-like Synoviocytes in rheumatoid arthritis. J Immunol Res..

[32] Wakabayashi K, Lian ZX, Leung PS, Moritoki Y, Tsuneyama K, Kurth MJ (2008). Loss of tolerance in C57BL/6 mice to the autoantigen E2 subunit of pyruvate dehydrogenase by a xenobiotic with ensuing biliary ductular disease. Hepatology.

[33] Wu SJ, Yang YH, Tsuneyama K, Leung PS, Illarionov P, Gershwin ME (2011). Innate immunity and primary biliary cirrhosis: activated invariant natural killer T cells exacerbate murine autoimmune cholangitis and fibrosis. Hepatology.

[34] Luz-Crawford P, Noel D, Fernandez X, Khoury M, Figueroa F, Carrion F (2012). Mesenchymal stem cells repress Th17 molecular program through the PD-1 pathway. PLoS One.

[35] Yang M, Deng J, Liu Y, Ko KH, Wang X, Jiao Z (2012). IL-10-producing regulatory B10 cells ameliorate collagen-induced arthritis via suppressing Th17 cell generation. Am J Pathol..

[36] Kawata K, Tsuda M, Yang GX, Zhang W, Tanaka H, Tsuneyama K (2013). Identification of potential cytokine pathways for therapeutic intervention in murine primary biliary cirrhosis. PLoS One.

[37] Wang Y, Chen X, Cao W, Shi Y (2014). Plasticity of mesenchymal stem cells in immunomodulation: pathological and therapeutic implications. Nat Immunol..

[38] Gieseke F, Bohringer J, Bussolari R, Dominici M, Handgretinger R, Muller I (2010). Human multipotent mesenchymal stromal cells use galectin-1 to inhibit immune effector cells. Blood.

[39] Souza BSF, da Silva KN, Silva DN, Rocha VPC, Paredes BD, Azevedo CM (2017). Galectin-3 knockdown impairs survival, migration, and immunomodulatory actions of mesenchymal stromal cells in a mouse model of Chagas disease cardiomyopathy. Stem Cells Int..

[40] Oo YH, Shetty S, Adams DH (2010). The role of chemokines in the recruitment of lymphocytes to the liver. Dig Dis..

[41] Wang L, Wang FS, Chang C, Gershwin ME (2014). Breach of tolerance: primary biliary cirrhosis. Semin Liver Dis..

[42] Burr SP, Dazzi F, Garden OA (2013). Mesenchymal stromal cells and regulatory T cells: the yin and Yang of peripheral tolerance?. Immunol Cell Biol..

[43] Ren G, Zhang L, Zhao X, Xu G, Zhang Y, Roberts AI (2008). Mesenchymal stem cell-mediated immunosuppression occurs via concerted action of chemokines and nitric oxide. Cell Stem Cell.

[44] Sioud M (2011). New insights into mesenchymal stromal cell-mediated T-cell suppression through galectins. Scand J Immunol..

[45] John S, Mishra R (2016). Galectin-9: from cell biology to complex disease dynamics. J Biosci..

[46] Rabinovich GA, Toscano MA (2009). Turning ‘sweet’ on immunity: galectin-glycan interactions in immune tolerance and inflammation. Nat Rev Immunol..

[47] Gieseke F, Kruchen A, Tzaribachev N, Bentzien F, Dominici M, Muller I (2013). Proinflammatory stimuli induce galectin-9 in human mesenchymal stromal cells to suppress T-cell proliferation. Eur J Immunol..

[48] Ungerer C, Quade-Lyssy P, Radeke HH, Henschler R, Konigs C, Kohl U (2014). Galectin-9 is a suppressor of T and B cells and predicts the immune modulatory potential of mesenchymal stromal cell preparations. Stem Cells Dev..

[49] Gnecchi M, Zhang Z, Ni A, Dzau VJ (2008). Paracrine mechanisms in adult stem cell signaling and therapy. Circ Res..

[50] Ratajczak MZ, Kucia M, Jadczyk T, Greco NJ, Wojakowski W, Tendera M (2012). Pivotal role of paracrine effects in stem cell therapies in regenerative medicine: can we translate stem cell-secreted paracrine factors and microvesicles into better therapeutic strategies?. Leukemia.

[51] Duran JM, Makarewich CA, Sharp TE, Starosta T, Zhu F, Hoffman NE (2013). Bone-derived stem cells repair the heart after myocardial infarction through transdifferentiation and paracrine signaling mechanisms. Circ Res..

[52] Choi J, Selmi C, Leung PS, Kenny TP, Roskams T, Gershwin ME (2016). Chemokine and chemokine receptors in autoimmunity: the case of primary biliary cholangitis. Expert Rev Clin Immunol..

[53] Manousou P, Kolios G, Drygiannakis I, Koulentaki M, Pyrovolaki K, Voumvouraki A (2013). CXCR3 axis in patients with primary biliary cirrhosis: a possible novel mechanism of the effect of ursodeoxycholic acid. Clin Exp Immunol..

[54] Oo YH, Banz V, Kavanagh D, Liaskou E, Withers DR, Humphreys E (2012). CXCR3-dependent recruitment and CCR6-mediated positioning of Th-17 cells in the inflamed liver. J Hepatol..

[55] Shimoda S, Harada K, Niiro H, Yoshizumi T, Soejima Y, Taketomi A (2008). Biliary epithelial cells and primary biliary cirrhosis: the role of liver-infiltrating mononuclear cells. Hepatology.

[56] Platanias LC (2005). Mechanisms of type-I- and type-II-interferon-mediated signalling. Nat Rev Immunol..

