# Supplementary material for: Mesenchymal stem cells alleviate experimental autoimmune cholangitis through immunosuppression and cytoprotective function mediated by galectin-9
Source: Stem Cell Res Ther. 2018 Sep 17;9:237. doi: 10.1186/s13287-018-0979-x (PMC6142687; doi:10.1186/s13287-018-0979-x)
Supplement: Supplementary file 1 — Figure S1. Umbilical cord–derived mesenchymal stem cell transplantation does not influence Th2 cells. n = 6; Bars represent the mean ± SEM; Abbreviations: PB peripheral blood, SP spleen. Figure S2. Umbilical cord–derived mesenchymal stem cell transplantation downregulates Th1/Th2 ratio. n = 6; Bars represent the mean ± SEM; *P <0.05, **P <0.01. Abbreviations: PB peripheral blood, SP spleen. Figure S3. Umbilical cord–derived mesenchymal stem cell transplantation does not affect the Th1 and Th17 immune responses in normal mice. (a, b) The alterations of Th1 and Th17 cells in different groups. n = 4; Bars represent the mean ± SEM; Abbreviations: PB peripheral blood, SP spleen. Figure S4. Umbilical cord–derived mesenchymal stem cell transplantation upregulates the expression of galectin-9 (Gal-9). a The serum levels of Gal-9 in different groups. n = 6. b The liver expression of Gal-9 in different groups. n = 3; Bars represent the mean ± SEM; **P <0.01, ***P <0.001. Abbreviation: GAPDH glyceraldehyde 3-phosphate dehydrogenase. Figure S5. Galectin-9(Gal-9) contributes to the immunoregulatory function of umbilical cord–derived mesenchymal stem cell conditioned media (MSC-CM). (a-c) The proliferation of CD4+ T cells, the alterations of Th1 and Th17 cells in different groups. n = 4; Bars represent the mean ± SEM; *P <0.05, **P <0.01, ***P <0.001. Abbreviations: CFSE Carboxyfluorescein diacetate succinimidyl ester. Figure S6. Umbilical cord–derived mesenchymal stem cell transplantation upregulates the regulatory T (Treg) cells. The alterations of Treg cells in different groups. n = 6; Bars represent the mean ± SEM; *P <0.05, **P <0.01, ***P <0.001. Abbreviations: PB peripheral blood, SP spleen. Figure S7. Specific staining of interferon-gamma (IFN-γ) in the liver section. Scale bar = 32 μm. Figure S8. Dynamic changes of engraftment umbilical cord–derived mesenchymal stem cell (UC-MSC) and galectin-9 (Gal-9) in the liver section. Scale bar = 50 μm. Abbreviation: GFP+ green flu [file 13287_2018_979_MOESM1_ESM.docx]

**Supplementary Figures**


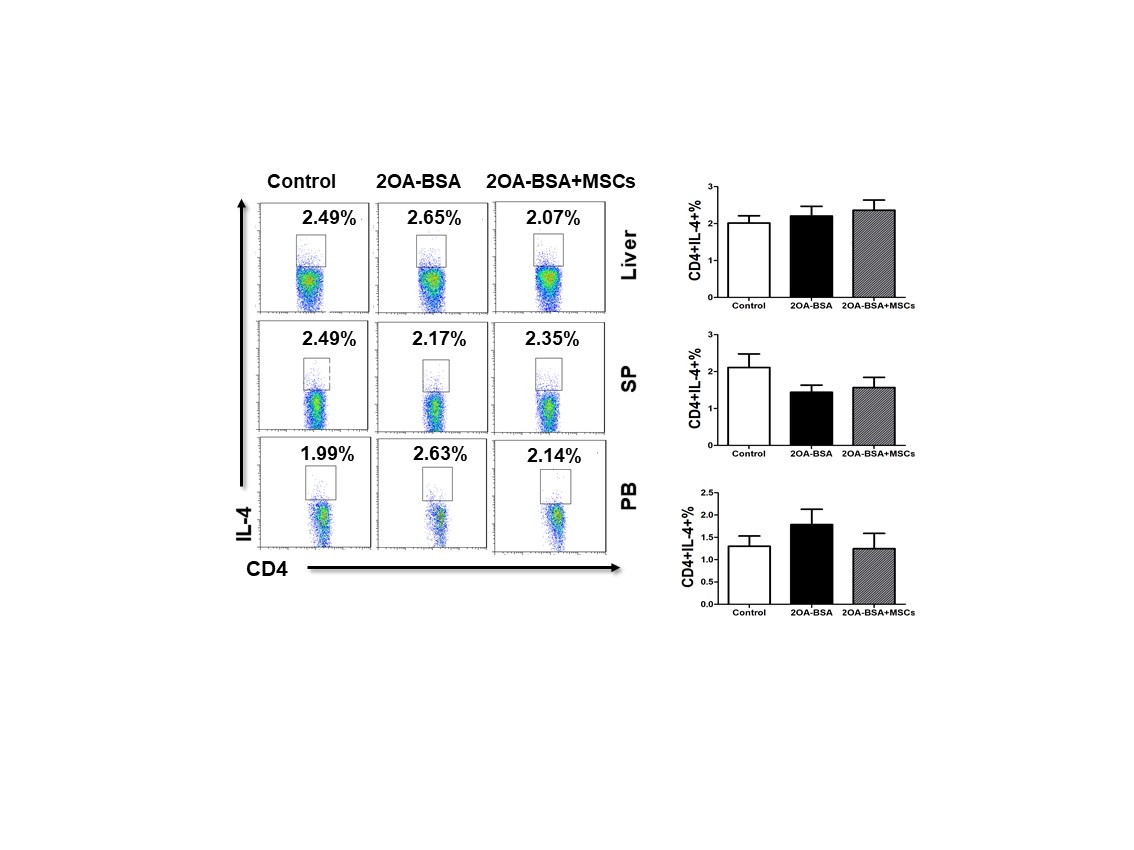


Figure S1 UC-MSCT does not influence Th2 cells in 2OA-BSA-induced autoimmune cholangitis. Representative flow cytometric profiles show the frequencies of Th2 cells in the liver, spleen and blood. The frequencies of Th2 cells were quantified. Bars represent the mean ± SEM; *n* = 6 per group. SP, spleen; PB, peripheral blood.


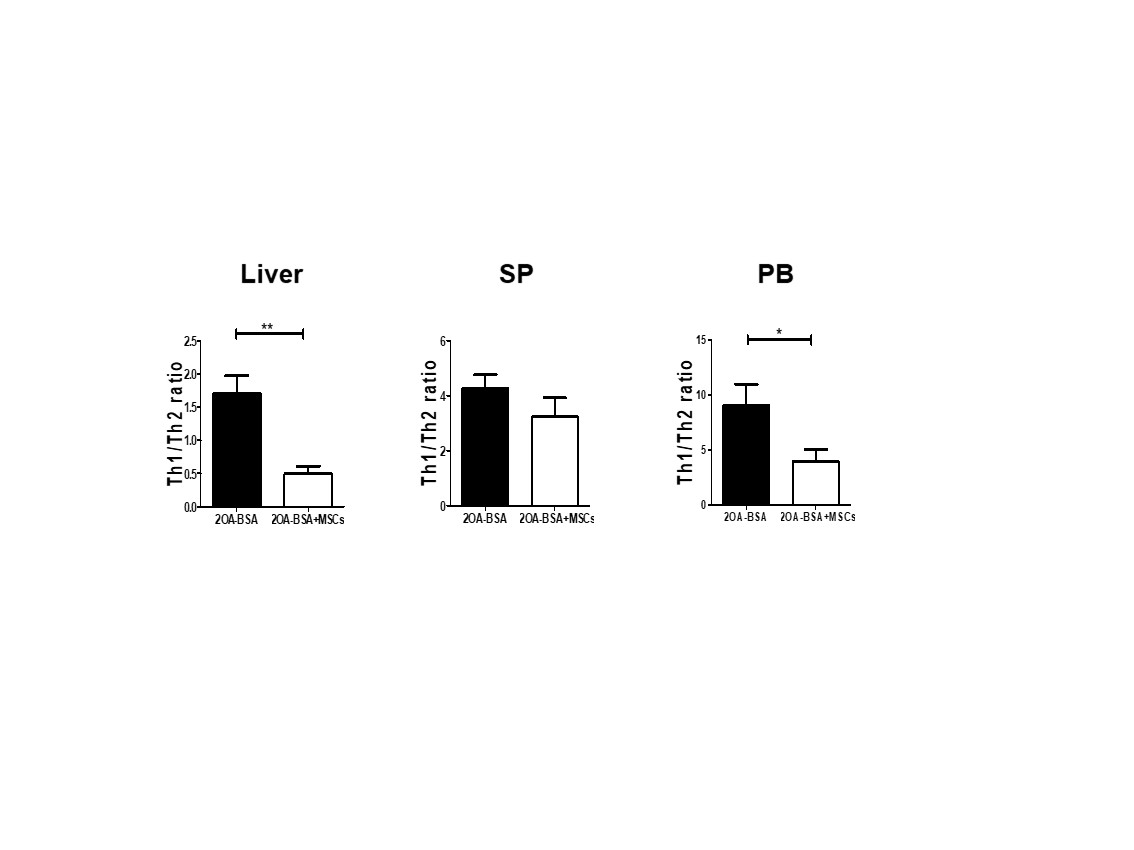


Figure S2 UC-MSCT downregulates Th1/Th2 ratio in 2OA-BSA-induced autoimmune cholangitis. Bars represent the mean ± SEM; *n* = 6 per group; **P*＜0.05, ***P*＜0.01. SP, spleen; PB, peripheral blood.


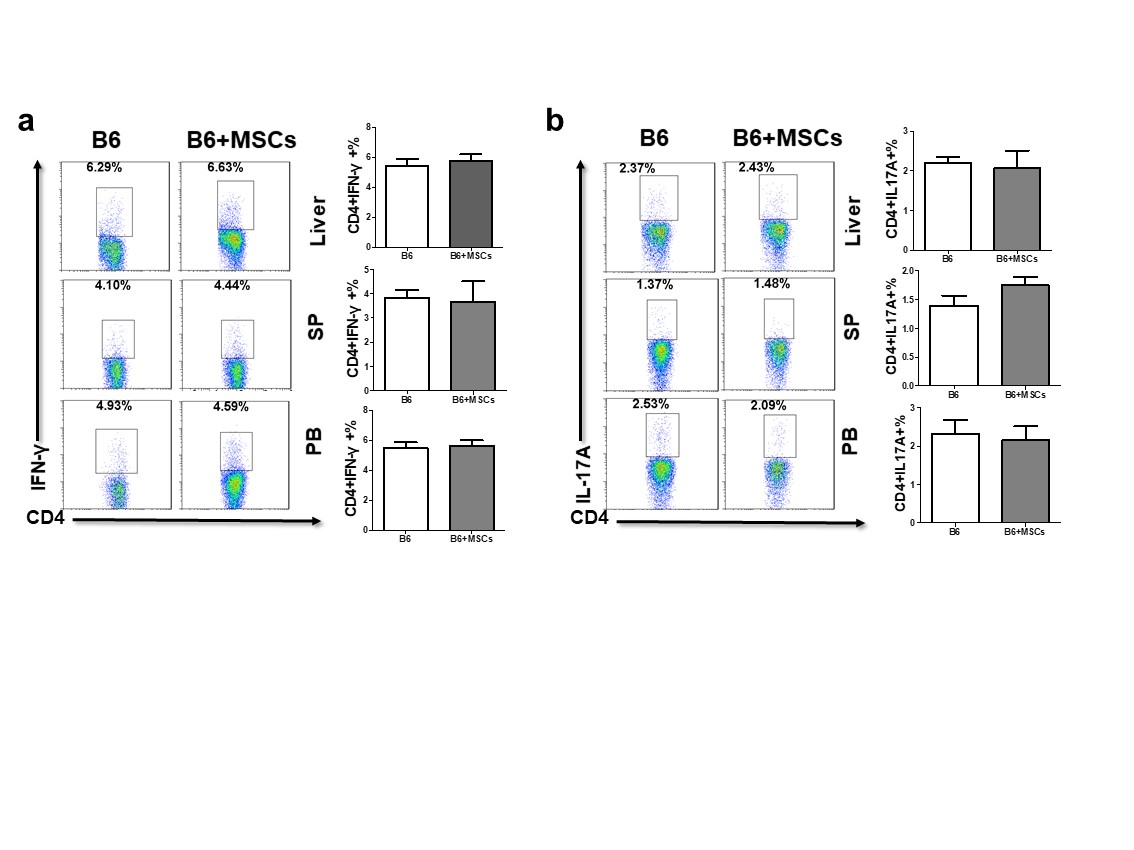


Figure **S3** UC-MSCT does not affect the Th1 and Th17 immune responses in normal C57BL/6 mice. **a** The Representative flow cytometric profiles and frequencies of Th1 cells in different groups. **b** The Representative flow cytometric profiles and frequencies of Th17 cells in different groups. Bars represent the mean ± SEM; *n*= 4 per group; SP, spleen; PB, peripheral blood.


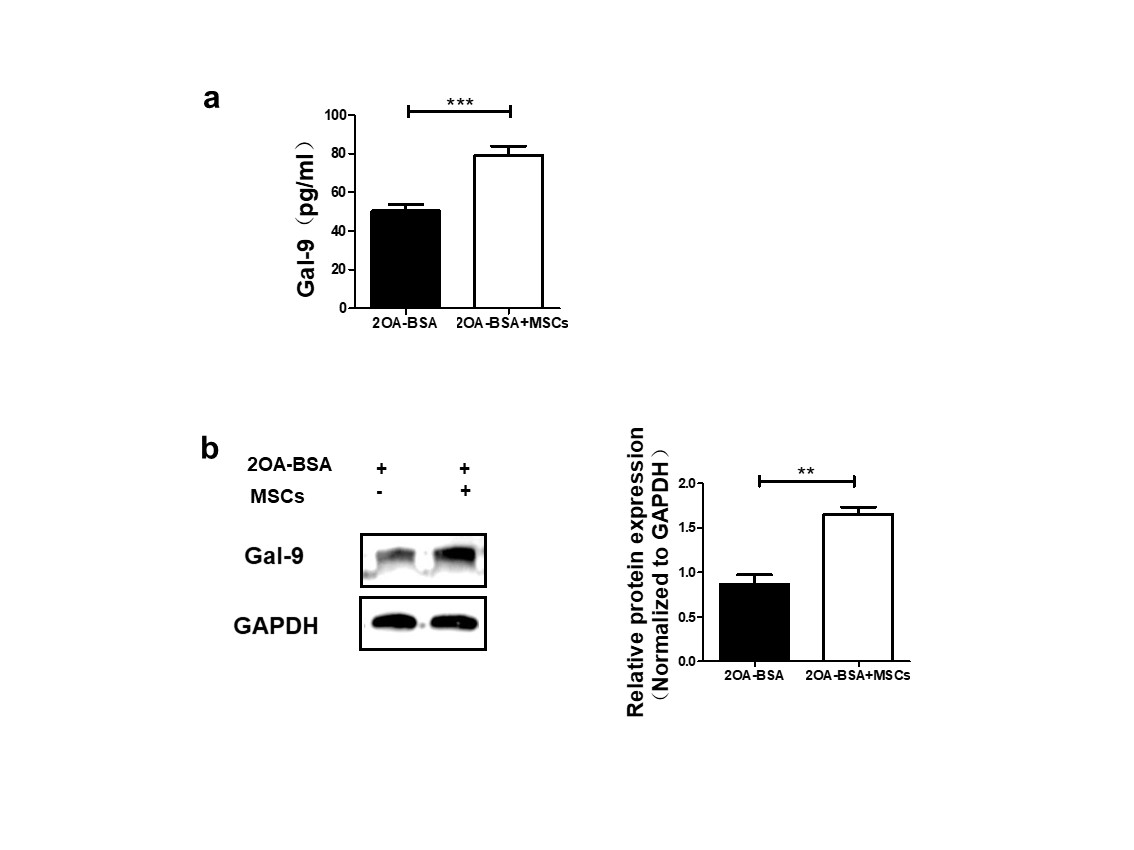


Figure **S4** UC-MSCT upregulates the expression of Gal-9 in 2OA-BSA-induced autoimmune cholangitis. **a** The serum levels of Gal-9 in different groups. Bars represent the mean ± SEM; *n*= 6 per group. **b** The liver expression of Gal-9 in different groups. Bars represent the mean ± SEM; *n*= 3 per group; ***P*＜0.01, ****P*< 0.001.


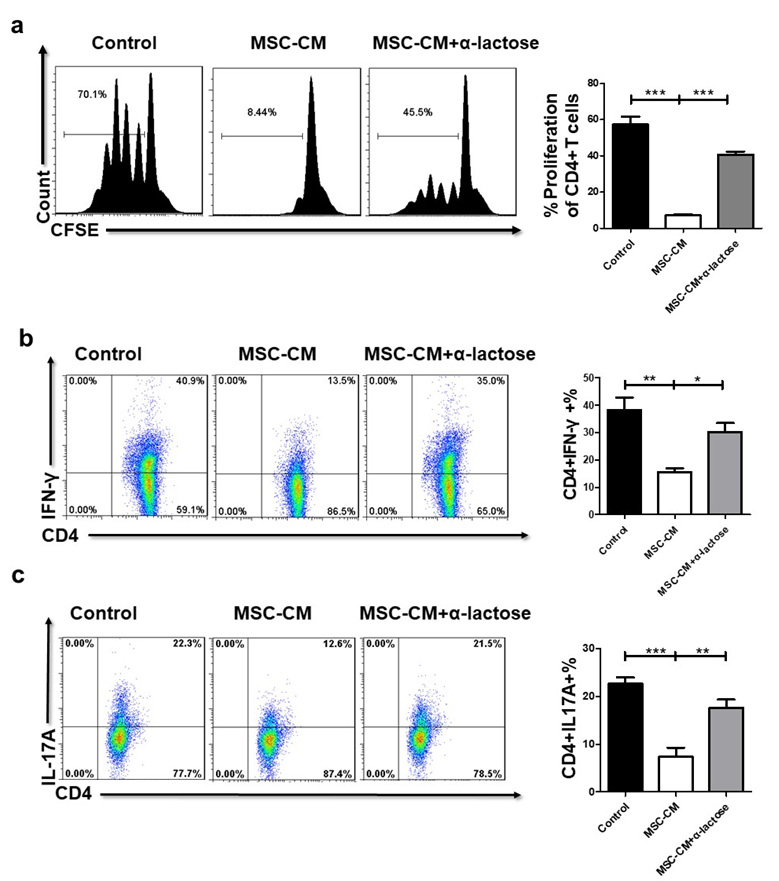


Figure **S5** UC-MSCs conditioned media (MSC-CM) suppresses CD4+T cells proliferation as well as Th1 and Th17 cell differentiation via Gal-9. **a** CFSE-labeled CD4+T cells were cultured in the presence of MSC-CM or MSC-CM plus α-lactose for 4 days. The proliferation of murine CD4+ T cells was assessed based on the fluorescence intensity of CFSE. Bars represent the mean ± SEM; *n*= 4 per group. **b** Purified naive murine CD4+ T cells were cultured under Th1 polarization conditions in the presence of MSC-CM or MSC-CM plus α-lactose. Representative flow cytometric profiles that show the frequencies of Th1 cells are presented. The frequencies of Th1 cells were quantified. **c** Purified naive murine CD4+ T cells were cultured under Th17 polarization conditions in the presence of MSC-CM or MSC-CM plus α-lactose. Representative flow cytometric profiles that show the frequencies of Th17 cells are presented. The frequencies of Th17 cells were quantified. Bars represent the mean ± SEM; *n* = 4 per group; **P*＜0.05, ***P*＜0.01, ****P*< 0.001.


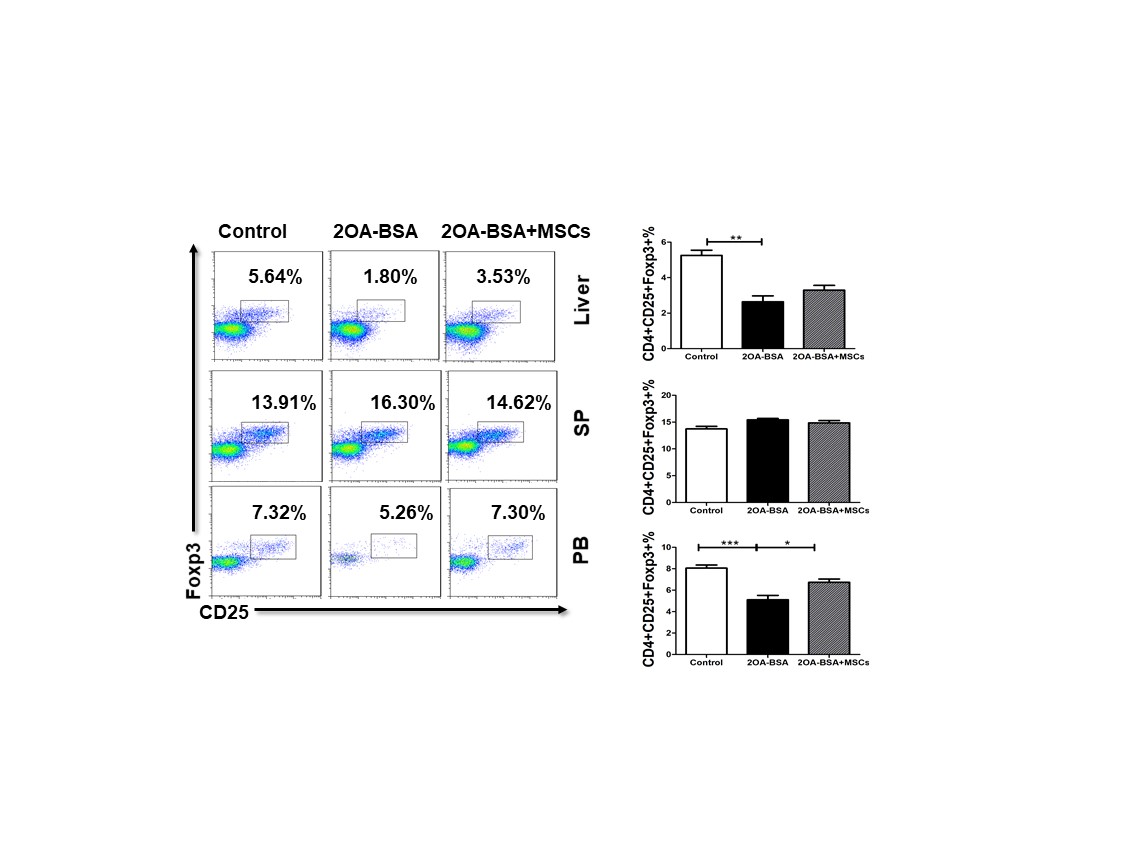


Figure **S6** UC-MSCT upregulates the frequencies of Treg cells from the peripheral blood in 2OA-BSA-induced autoimmune cholangitis. The representative flow cytometric profiles and frequencies of Treg cells in different groups. Bars represent the mean ± SEM; *n*= 6 per group; SP, spleen; PB, peripheral blood; **P*＜0.05, ***P*＜0.01, ****P*< 0.001.


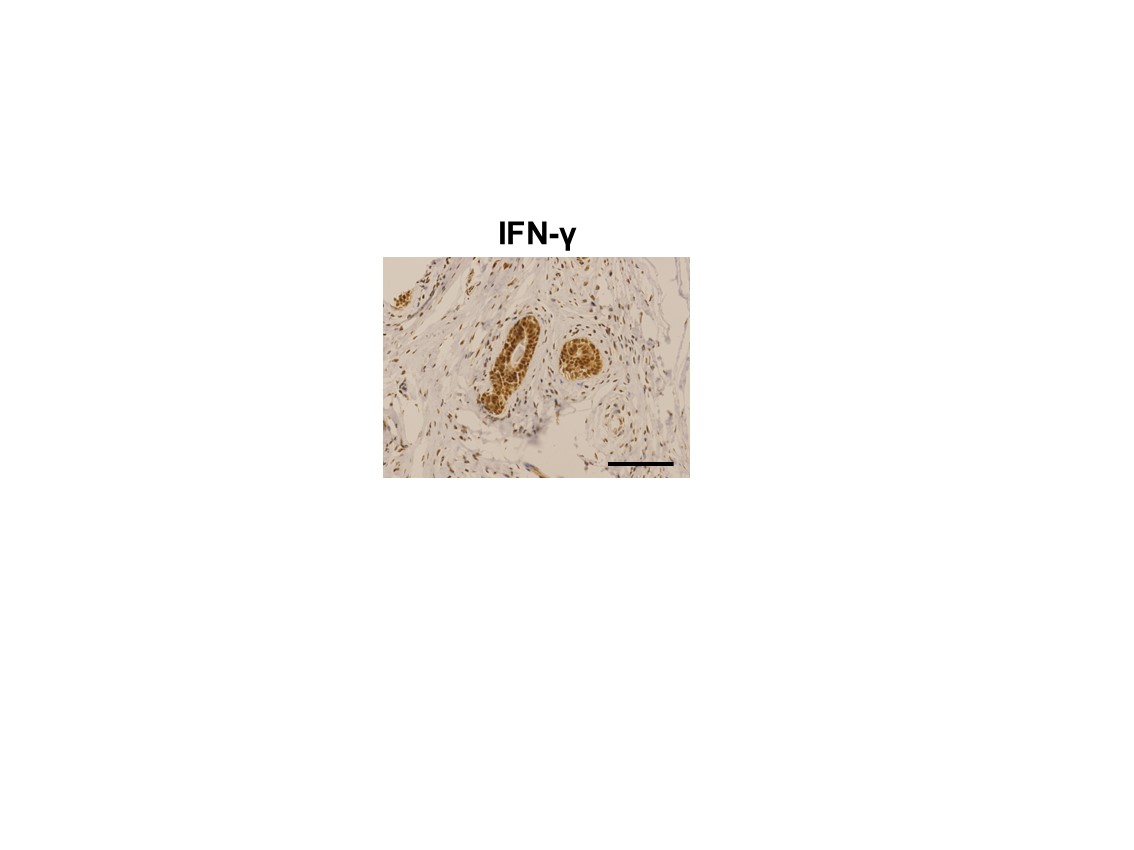


Figure **S7** Specific staining of INF-γ in the liver section of 2OA-BSA-induced autoimmune cholangitis. Scale bar = 32 μm.


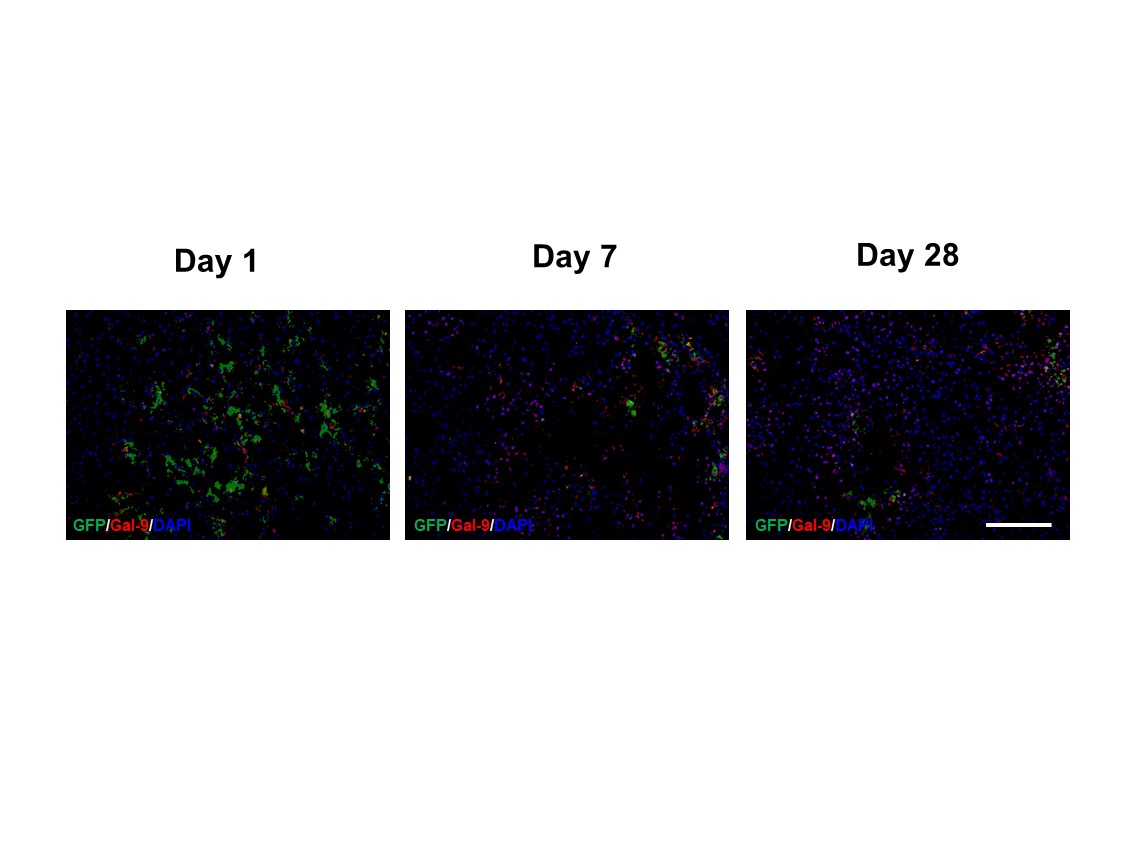


Figure **S8** Dynamic changes of engraftment UC-MSCs and Gal-9 in the liver section of 2OA-BSA-induced autoimmune cholangitis. At diﬀerent time points, PBC model mice were sacrificed and frozen section of the liver tissue was made. The ﬂuorescence microscope were employed to detect GFP ^+^(green) UC-MSCs and Gal-9^+^ (red) in the liver section. Scale bar = 50 μm.
