# Supplementary material for: Mesenchymal stem cells alleviate experimental autoimmune cholangitis through immunosuppression and cytoprotective function mediated by galectin-9
Source: Stem Cell Res Ther. 2018 Sep 17;9:237. doi: 10.1186/s13287-018-0979-x (PMC6142687; doi:10.1186/s13287-018-0979-x)
Supplement: Supplementary file 2 — Supplementary Materials and Methods. (DOCX 21 kb) [file 13287_2018_979_MOESM2_ESM.docx]

**Supplementary Materials and Methods**

**Induction of cholangitis and** **UC-MSCT**

For the induction of autoimmune cholangitis, 100 μg 2OA-BSA conjugate (in 50 μl PBS) was emulsified with 50 μl of complete Freund's adjuvant (CFA; containing 1mg/ml of mycobacterium tuberculosis strain H37RA, Sigma-Aldrich) and injected intraperitoneally (I.P.) into female C57BL/6 mice. Additionally, mice received 100 ng of pertussis toxin (List Biological Laboratories, Campbell, CA, USA) in 100 μl PBS by I.P. at the time of initial immunization with 2OA-BSA and two days afterwards, respectively. 2 weeks later, the mice were re-boosted I.P. with 100 μg 2OA-BSA in 50 μl PBS emulsified with 50 μl of incomplete Freund's adjuvant (IFA; Sigma-Aldrich). The treatment of UC-MSCT in C57BL/6 mice and 2OA-BSA immunized mice were performed. Mice were injected intravenously with 1× 10^6^ UC-MSCs and then sacrificed at 12 weeks post immunization.

**Cell preparation and flow cytometry analysis**

Livers, spleens and peripheral blood were harvested immediately following sacrifice of the mice. Livers were first perfused with PBS containing 0.2% BSA (0.2% BSA/PBS), passed through a 100 μm nylon cell strainer and re-suspended in 0.2% BSA/PBS. Hepatocytes were removed as pellets after centrifugation at 75 g for 1 min and the remaining suspended cells were collected. Spleens were disrupted between two glass slides and suspended in 0.2% BSA/PBS. Mononuclear cells (MNCs) from the livers were isolated by gradient centrifugation using 40% and 70% Percoll (Sigma-Aldrich). Peripheral blood mononuclear cells **(**PBMCs) were obtained by lysis of erythrocytes in the blood. The following antibodies were used: anti-CD4, anti-IL-4, anti- INF-γ, anti-IL-17A, anti-CD25 and anti-Foxp3 (eBioscience, San Diego, CA, USA). For intracellular cytokine staining, cells were stimulated with 20 ng/ml phorbol-12-myristate-13-acetate plus 1 µg/ml ionomycin at 37°C for 4–5 h in the presence of 5 µg/ml brefeldin A (all from Enzo Life Science, Farmingdale, NY, USA). Then the cells were fixed and permeabilized with a fixation/permeabilization kit (Nordic-MUbio, Maastricht, Limburg, the Netherlands), followed by staining with anti-IL-4, anti-INF-γ and anti-IL-17A. Intranuclear staining of Foxp3 was performed using Foxp3/Transcription Factor Staining Buffer Set (eBioscience). Data were acquired by a FACS Calibur flow cytometer (BD Biosciences, Mountain View, CA, USA) and were analyzed with FlowJo software (Tree Star, Ashland, OR, USA).

**Isolation and culture of naïve CD4+ T cells**

For Th1 cell differentiation, naïve CD4+ T cells were purified from spleens using a naïve CD4+ T cell Isolation Kit (Stem Cell Technologies, Vancouver, Canada) and were cultured in 2.5 µg/ml anti-CD3 and 5 µg/ml anti-CD28 (eBioscience) pre-coated culture plates with 20 ng/ml IL-2 (PeproTech), 20 ng/ml IL-12 (PeproTech) and 5 µg/ml anti-IL-4 (eBioscience) for stimulation. For Th17 cell differentiation, naïve CD4+ T cells received the same treatment as for Th1 differentiation except for 20 ng/ml IL-6 (PeproTech), 3 ng/ml TGF-β (PeproTech), 20 ng/ml IL-23 (PeproTech), 5 µg/ml anti- IFN-γ (eBioscience) and 5 µg/ml anti- IL-4 (eBioscience) were used for stimulation instead. To confirm the role of Gal-9 in UC-MSCs, the cells were treated with conditioned media (CM) collected from UC-MSCs (MSC-CM) alone or MSC-CM plus 10.8 mg/ml α-lactose (Sigma-Aldrich). After culture for 72h, the cells were collected for further analysis.

**Proliferation assay**

Murine CD4+ T cells were purified from spleens according to the manufacturer’s instruction (BD Biosciences). For the proliferation assay, CD4+ T cells at the density of 1×10^6^ cells /well were labeled with 5 μM carboxyﬂuorescein diacetate succinimidyl ester (CFSE, Invitrogen, Camarillo, CA, USA), and then cocultured with MSC-CM or MSC-CM with 10.8 mg/ml α-lactose (Sigma-Aldrich) for 4 days. Cell division was determined by measuring the CFSE fluorescence intensity by flow cytometry.

**Enzyme-linked immunosorbent assay**

The serum levels of Gal-9 (Cloud-Clone Corp, Houston, Texas, USA) in different groups were measured by ELISA kits. The procedures were performed according to the manufacturer’s instructions.

**Western blot assay**

For western blot assay, rabbit anti-Gal-9 antibody (Proteintech, Wuhan, Hubei, P.R. China), and mouse anti-GAPDH antibody (Proteintech) were used as primary antibodies. Horseradish peroxidase (HRP)-conjugated anti-rabbit IgG (H+L) and anti-mouse IgG (H +L) (Proteintech) were used as secondary antibodies, respectively. UC-MSCs were washed twice with PBS and lysed on ice for 30 min with 1× radioimmunoprecipitation assay (RIPA) buffer (CST) containing 1% 100× protease/phosphatase inhibitor cocktail (CST). Lysates were centrifuged at 12,000 *g* at 4°C for 20 min, and then the supernatants were subjected to sodium dodecyl sulfate polyacrylamide gel electrophoresis (SDS-PAGE). Protein was then transferred to polyvinylidene fluoride membranes (Millipore), blocked for 1 h in 5% nonfat milk in TBST [10 mM Tris (BioSharp, Hefei, Anhui, P.R. China) (pH 7) and 150 mM NaCl, 0.1% Tween 20], and then immunoblotted with the above-listed primary antibodies and appropriate HRP conjugated secondary antibodies. Chemiluminescence HRP substrate (Millipore) was used to detect the specific proteins, and the bands were visualized using the G:BOX gel imaging system (Syngene, Cambridge, UK). Analysis was performed using ImageJ software (NIH, Bethesda, MD, USA).

**Immunohistochemistry**

For immunohistochemistry, paraffin sections of the liver were incubated with primary rat anti-INF-γ followed by appropriate secondary antibodies incubation. The immunostaining was developed using diaminobenzidine tetrahydrochloride, followed by counterstain with Mayer’s Hematoxylin.

**Cells labeling with GFP**

To track the transplanted cells *in vivo*, UC-MSCs were labeled with GFP by lentivirus infection. Briefly, the pLV-CMV-GFP-Neo vector, PMD2.G and PSPAX2 packaging plasmids, and the X-treme GENE HP DNA Transfection Reagent (Roche, Basel, Switzerland) were added to 10% FBS DMEM medium, mixed gently, and incubated at room temperature for 20 min. The mixture was added dropwise into 293T cells in a 10cm plate. After 48h incubation, the virus supernatant was collected and filtered using a 0.45-mm filter. UC-MSCs were then infected with the virus. After being cocultured for 48 h, the aminoglycoside antibiotic G418 (Gibco-BRL, Carlsbad, CA, USA) was added to the medium at a final concentration of 600 mg/mL to select UC-MSCs with a stable GFP expression. The UC-MSCs labeled with GFP were observed with a fluorescence emission ratio at 530 nm using an epifluorescence microscope and an excitation wavelength of 488 nm.

**Immunoﬂuorescence**

For the immunoﬂuorescence examination, liver tissues were OCT-embedded, snap-frozen, and cut into 3 μm slides. Then slides were incubated with primary antibody overnight at 4 °C. Primary antibodies were as follows: Gal-9 (1:200; abcam, USA). Slides were stained with DAPI and then examined by ﬂuorescence microscope. The scale bar of the picture at 200 magnification represent 50μm.

**Statistical analysis**

Data are presented as the mean ± standard error of the mean (SEM). Student’s *t*-test was applied when two groups were compared for significant differences. A one-way analysis of variance (ANOVA) followed by the Newman–Keuls test was used to compare more than two groups. The Prism statistical package (Graph Pad Software Inc, La Jolla, CA, USA) was used. A *P*-value < 0.05 was considered statistically significant.
